# Improving the Therapeutic Ability of Mesenchymal Stem/Stromal Cells for the Treatment of Conditions Influenced by Immune Cells

**DOI:** 10.1155/2019/6820395

**Published:** 2019-12-06

**Authors:** Lindolfo da Silva Meirelles, Marcela F. Bolontrade, Melissa Medeiros Markoski, Bruno Dallagiovanna, Laura Alaniz

**Affiliations:** ^1^PPGBioSaúde, Lutheran University of Brazil, Canoas, Brazil; ^2^IMTIB-CONICET-IUHI-HIBA, Buenos Aires, Argentina; ^3^Federal University of Health Sciences of Porto Alegre, Porto Alegre, Brazil; ^4^Carlos Chagas Institute–Fiocruz Paraná, Curitiba, Brazil; ^5^CIT NOBA–CONICET-UNNOBA, Junín, Argentina

Mesenchymal stem/stromal cells (MSCs) have been initially described decades ago as fibroblastic precursors that could be isolated from the bone marrow and establish cultures of fibroblastic cells. These fibroblastic cells were shown to support hematopoiesis in vitro, which is a characteristic of stromal cells, and, later, to give rise to mature mesenchymal cells such as bone, cartilage, and fat cells when cultured under appropriate conditions. The proposition that a mesenchymal stem cell exists in postnatal bone marrow and other tissues as blood vessel-associated cells provided further momentum to research on these cells, as well as divergences on how to call them. The impetus of using MSCs to replace cells lost in various types of conditions eventually decreased, as the therapeutic benefits provided by these cells were found to be mostly due to the secretion of paracrine signaling molecules, which can be carried by extracellular vesicles. In the meantime, MSCs were found to modulate the behavior of immune cells by means of secretion of molecules that could, in different scenarios, inhibit the activation of T cells that promote adaptive immune responses. Subsequently, the effects of MSCs on other cells of the immune system were also described. Today, a number of clinical trials using MSCs to treat conditions influenced by immune cells are under way. While preclinical data indicates that MSCs have important immunomodulatory properties, further studies are still in progress to increase the knowledge on the differences regarding the action of MSCs on immune cells according to their tissue of origin, on how MSCs exert their effects on the different types of immune cells, and on ways to improve the outcome of conditions influenced by immune cells when treated using MSCS.

This special issue was open to basic research manuscripts and reviews that approached ways to improve the therapeutic ability of MSCs for the treatment of conditions influenced by immune cells. Accordingly, two basic research papers on the interactions between MSCs and immune cells in skin wound models and three reviews on aspects of the relations between these cells were accepted. S. Xiao et al. showed that forced expression of interleukin-10, an anti-inflammatory cytokine, in human amnion-derived MSCs improves the healing of full-thickness skin wounds in mice by reducing inflammation and excessive extracellular matrix deposition while improving angiogenesis. He and his colleagues focused on the effects of exosomes, a type of extracellular vesicle, produced by MSCs isolated from human bone marrow on the polarization of macrophages in full-thickness skin wounds of mice. Consequently, X. He et al. found that microRNA-223 present in these exosomes contribute to a proregenerative M2 polarization in macrophages. B. S. Guerrouahen et al. reviewed mechanisms underlying communication of MSCs with immune cells and discussed clinical applications of MSCs in diseases mediated by immune cells. F.V. Paladino et al. reviewed the immunomodulatory properties of Wharton's jelly-derived MSCs. Finally, Z. Fábián discussed the effects of hypoxia on the immunomodulatory properties of the bone marrow. This group of articles provides a contribution to better understand and, consequently, improve the therapeutic properties of MSCs for the treatment of conditions influenced by the immune system.

